# Network analysis of preictal iEEG reveals changes in network structure preceding seizure onset

**DOI:** 10.1038/s41598-022-16877-x

**Published:** 2022-07-22

**Authors:** Stefan Sumsky, L. John Greenfield

**Affiliations:** 1 Department of Neurology, University of Connecticut, Farmington, CT 06070 USA; 2 Department of Neurology, UConn Health, 263 Farmington Avenue, Farmington, CT 06030-5357 USA

**Keywords:** Neural circuits, Neuronal physiology, Epilepsy, Biomedical engineering

## Abstract

Seizures likely result from aberrant network activity and synchronization. Changes in brain network connectivity may underlie seizure onset. We used a novel method of rapid network model estimation from intracranial electroencephalography (iEEG) data to characterize pre-ictal changes in network structure prior to seizure onset. We analyzed iEEG data from 20 patients from the iEEG.org database. Using 10 s epochs sliding by 1 s intervals, a multiple input, single output (MISO) state space model was estimated for each output channel and time point with all other channels as inputs, generating sequential directed network graphs of channel connectivity. These networks were assessed using degree and betweenness centrality. Both degree and betweenness increased at seizure onset zone (SOZ) channels 37.0 ± 2.8 s before seizure onset. Degree rose in all channels 8.2 ± 2.2 s prior to seizure onset, with increasing connections between the SOZ and surrounding channels. Interictal networks showed low and stable connectivity. A novel MISO model-based network estimation method identified changes in brain network structure just prior to seizure onset. Increased connectivity was initially isolated within the SOZ and spread to non-SOZ channels before electrographic seizure onset. Such models could help confirm localization of SOZ regions.

## Introduction

The need to accurately identify the seizure onset zone (SOZ) for epilepsy surgery has led to intense interest in characterizing the SOZ and mechanisms of seizure initiation and propagation. Mapping of the epileptic network shows promise as a powerful tool for understanding these processes^1,2^ and could help target tissues for resection to ensure a seizure-free outcome with the least damage to normal brain function.

Functional and effective epileptic networks represent time-dependent connections between neuronal populations that have been damaged, malformed, or functionally misconnected, rendering the brain vulnerable to seizure generation or spread. Early studies characterized the structure of the seizure network as highly synchronous^3–5^, but more recent work provides evidence that more complex changes in the brain network structures may be critical to seizure generation^6–11^.

Most prior investigations of dynamic brain networks have used functional connectivity techniques such as correlation or cross-correlation, coherence, phase synchronization, phase-slope, or Granger causality. Of these approaches, only cross-correlation and Granger causality can provide directed connectivity, and all of them are dependent on shared signal properties in the time or frequency domains^12,13^. This contrasts with effective connectivity, which depends on state space models with connections that are not merely directed, but causal in nature^14^. Information about directed causal influence in brain networks may be critical to understanding how seizures arise and propagate^15,16^. Existing methods of estimating effective connectivity are often problematic for electrophysiological brain recordings. For example, dynamic causal modeling requires advanced Bayesian selection of a prior model^17,18^, while structural equation modeling is inherently unstable when applied to time series data^19^. Neither method is suitable for investigating the effective network properties at seizure onset.

In this study, we propose a novel methodology for effective network estimation that utilizes multiple-input–single-output (MISO) state-space modeling of electrophysiological data from intracranial electroencephalographic (iEEG) recordings. This modeling approach allows the capture of the dominant multi-region network dynamics underlying the observed iEEG activity by directed network identification from short (10 s) recording epochs, enabling study of the temporal evolution of the seizure network and the directed flow of information within that network. We measured changes in degree centrality (DC, the number of connections at a given node), and betweenness centrality (BC, the number of shortest paths through a given node)^20^. These measures quantify the level of interconnectivity in the network and the extent to which specific nodes are critical to signal spread and information flow, both of which have high relevance for understanding seizure dynamics and can inform models of seizure mechanisms. In this instance, network nodes are the electrode contacts of intracranial electrodes, representing the loci of summed local field potentials at that site, and edges represent the anatomical and physiological interconnections with other neuronal regions (nodes). We examined the dependence of the signal at each node on the signal present at every other node, and how that dependence changes over time. Our results indicate a succession of directed network state changes just prior to the electrographic seizure onset, which suggest a pattern of underlying physiological changes associated with seizure initiation.

## Results

### Network analysis

For each of the 143 seizures studied, 120 s of iEEG data immediately prior to seizure onset were divided into 120 ten-second epochs using a sliding 1-s window and used to estimate sequential directed preictal network structures. Similar networks were subsequently generated for the continuous interictal data (excluding the period two hours before and after each seizure) for all patients using the same process for comparison.

### Preictal changes in degree and betweenness centrality

**Figure 1:**
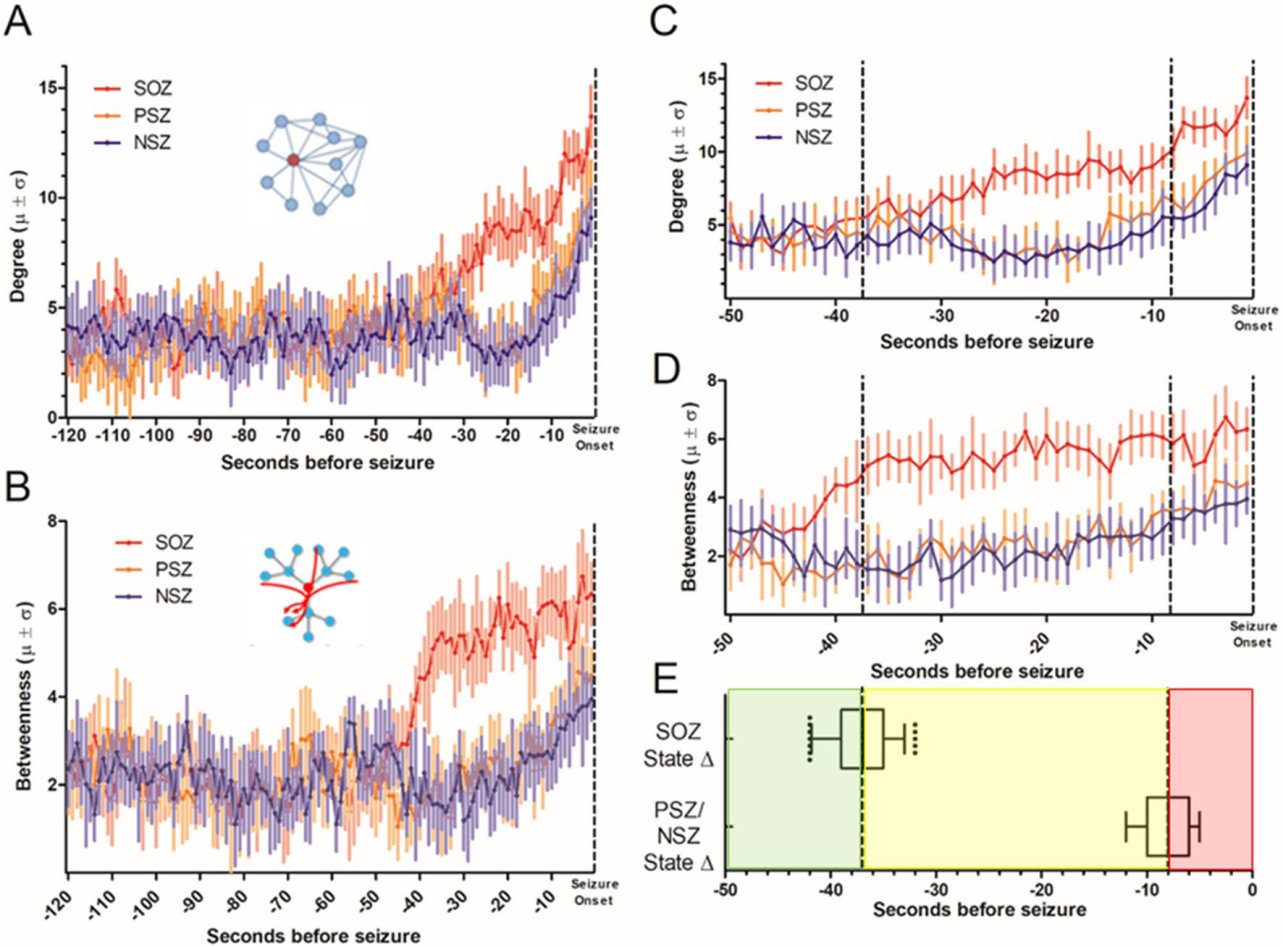
Preictal network centrality 2 min prior to seizure onset. Models were generated using a sliding 10 s window at 1 s intervals. (**A**) Average degree centrality (DC) of preictal SOZ (mean = red dots and solid line, ± standard deviation (SD) in lighter red) PSZ (orange dots and solid line, SD in lighter orange) and NSZ (blue dots and solid line, SD in lighter blue). Symbols appear at the onset of the reported period. The period from − 50 s to seizure onset is shown at greater resolution in part (**C**). (**B**) Betweenness centrality (BC) for the same period and contact groups, shown at greater resolution in part (**D**). (**E**) Transition time between network connectivity states determined by shifting the 10 s epoch by 1 s intervals for each seizure to determine the epoch when degree centrality was 1 standard deviation above the previous state value. The increase in SOZ BC (SOZ ∆) occurred at − 37.0 ± 2.8 s (mean ± SD, median − 37 s), and the change at NSZ nodes (PSZ/NSZ ∆) occurred at − 8.2 ± 2.2 s (median − 8 s). Boxes show 25th to 75th %ile around the median; whiskers are at 5% and 95%. Elevations in DC and BC were significant (Kruskal Wallis with post-hoc Dunn’s test, p < 0.05) for at most points for SOZ after the first transition and for PSZ and NSZ after the second transition, but are not shown for clarity.

The directed nature of the estimated network structures was used to investigate changes in connectivity over the preictal time period. From 120 to about 40 s prior to seizure onset, there was low overall degree centrality (DC, the number of connections made to each node, Fig. 1A). Subsequent analysis of the entire interictal period (excluding the 2 h before and after each seizure) demonstrated no significant difference in DC compared to that seen during the − 120 to − 40 s time points (see below). At about − 40 s and after, SOZ contacts show progressively increasing DC, rising above the range of prior interictal SOZ connectivity, and also higher than the peri-seizure onset zone (PSZ) and non-seizure onset zone (NSZ) contacts for all time points from about − 40 s until seizure onset. During the final 10 s of the preictal period, there is an additional change in network connectivity, as PSZ and NSZ contacts also increase DC significantly above their interictal values (shown at greater time resolution in Fig. 1C).

Similarly, betweenness centrality (BC, the number of shortest paths through each node, Fig. 1B), is significantly higher in SOZ nodes at the time points from about − 40 to about − 10 s relative to SOZ during the interictal state, and higher than all PSZ and NSZ nodes during the same approximate − 40 to − 10 s time period, suggesting a distinct network state. There are then increases in BC for the other contacts: BC values at PSZ and NSZ contacts become significantly higher than interictal about 10 s prior to seizure onset (Fig. 1D). This apparent spread of increased connectivity from SOZ to PSZ and NSZ nodes around 10 s prior to seizure onset suggested a second distinct preictal state that differed from interictal levels. Hence, preliminary network analysis of the 2 min period prior to seizure onset revealed 3 time periods with differing connectivity properties consistent with distinct preictal network states:

1. the period from − 120 to about − 40 s, unchanged from the interictal state,
2. from − 40 to about − 10 s, when the SOZ network increases connectivity, while PSZ and NSZ contacts remain unchanged, and
3. from about − 10 s until seizure onset, when PSZ and NSZ networks increase connectivity.

### Timing and consistency of network state transitions

To determine with greater specificity the transition time points when these changes occur, we determined for each seizure the epoch in which BC value was 1 standard deviation (SD) above the previous state value, which was considered the state transition time, and then averaged these times across seizures to determine the transition mean and SD. Betweenness was used since the changes in this parameter appeared more robust and consistent than DC for early changes. The transition from connectivity levels prior to − 40 s to those seen in SOZ nodes after − 40 s occurred at − 37.0 ± 2.8 s (mean ± SD, median − 37 s), and the change in connectivity at PSZ and NSZ nodes after − 10 s occurred at − 8.2 ± 2.2 s (median − 8 s, Fig. 1E). These transition time points were used in all subsequent analyses. For convenience, we have labeled the − 120 to − 38 s epoch “Interictal,” since BC and DC did not differ from interictal values more distant from seizure onset (see below). We labeled the network state after the first state transition “SOZ ∆” and the state after the second state transition “PSZ/NSZ ∆”.

**Figure 2:**
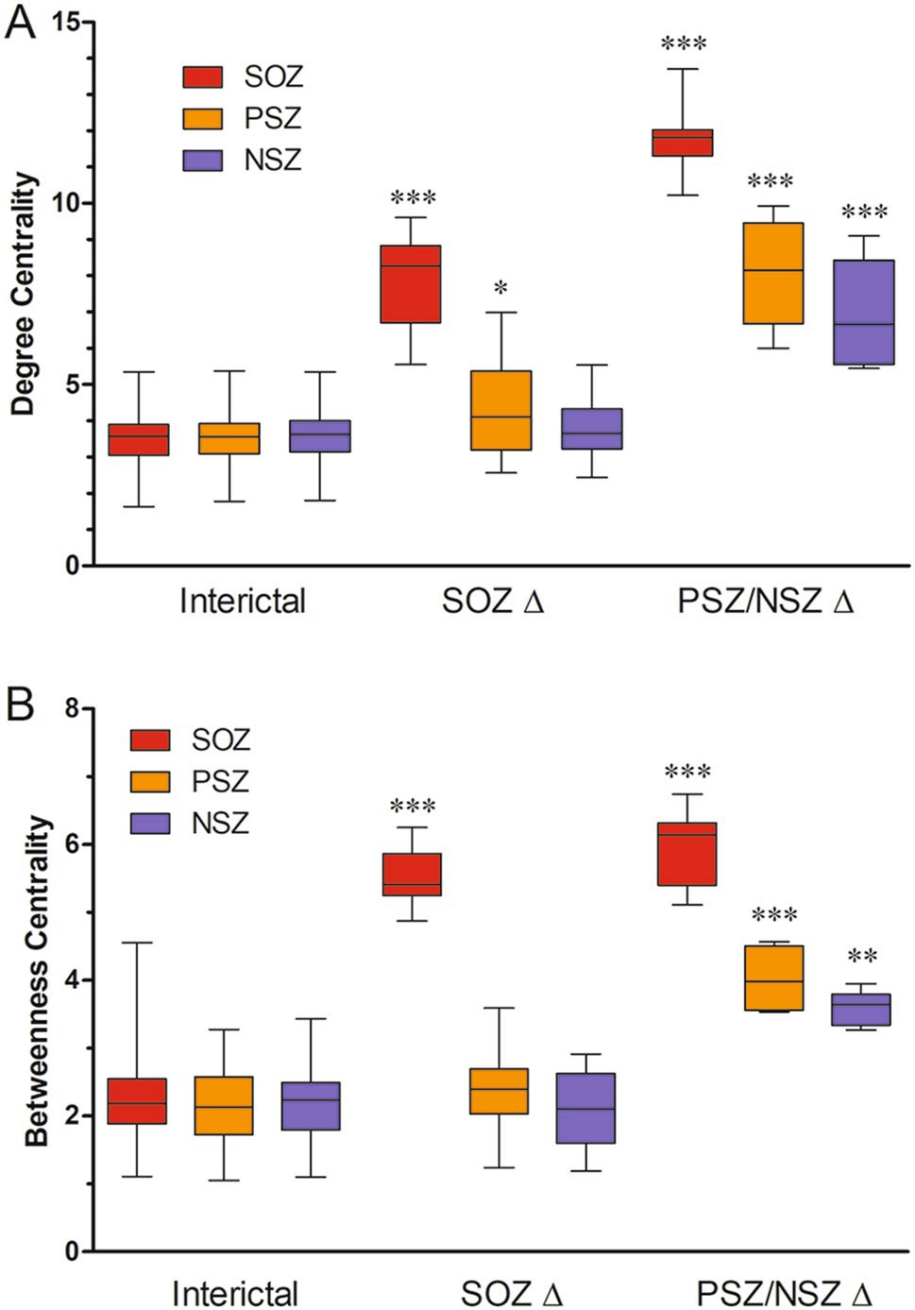
Network centrality measures during preictal states. (**A**) Average degree centrality (DC) of SOZ, PSZ, and NSZ channels during the interictal state, the state from − 37 to − 9 s (after SOZ ∆ and prior to PSZ/NSZ ∆, labeled SOZ ∆), and the state from − 8 s until − 1 s before seizure onset (after PSZ/NSZ ∆). (**B**) Average betweenness centrality (BC) for the same channel groups during the same periods before seizure. The interictal data for both plots include the entire interictal period (excluding the 2 h before and after a seizure), and were unchanged from the − 120 to − 39 s period. Boxes represent 25th to 75th percentile; whiskers represent minimum and maximum values. Asterisks denote significant difference by Kruskal Wallis test with post-hoc Dunn’s test (***p < 0.001, **p < 0.01, *p < 0.05).

To assess the consistency of the changes in network interconnections within each putative time-dependent state, we determined the mean and range of DC (plotted as box and whiskers, Fig. 2A) and BC (Fig. 2B) for each of the three identified network states by averaging the network values at each 1 s time point within the putative states. For the interictal period, we included the entire interictal record modeled at 1 s intervals to ensure that there were no periods in which DC rose above the threshold seen in the immediate (− 120 to − 38 s) preictal period. There were only small differences in DC or BC connectivity between SOZ, PSZ or NSZ during the entire interictal period, and the range of DC and BC interictal values for all points at SOZ contacts did not overlap with the range during the SOZ ∆ period (in Fig. 2, the “whiskers” represent the entire range of values). In the SOZ ∆ epoch, both DC and BC were significantly elevated at SOZ electrodes (p < 0.001) relative to interictal SOZ and to PSZ and NSZ electrodes at the same time period. PSZ contacts had slightly but significantly (p < 0.05) higher DC than in the interictal period, though BC at PSZ contacts was unchanged. NSZ contacts had no change in either DC or BC relative to interictal values. In the PSZ/NSZ ∆ epoch, DC at the SOZ contacts was significantly (p < 0.001) higher than in the SOZ ∆ epoch (or the interictal period), and there were now significant increases in DC at the PSZ and NSZ contacts (both p < 0.001 relative to interictal). BC at SOZ contacts in the PSZ/NSZ ∆ epoch was also significantly elevated relative to interictal, but not significantly higher than in the SOZ ∆ epoch. BC was also elevated in the PSZ/NSZ ∆ epoch at PSZ (p < 0.001) and NSZ (p < 0.05) contacts relative to interictal. The consistency of network centrality characteristics within each of these defined periods, and their significant differences in DC and BC relative to the interictal period, confirm that they represent 3 distinct time-dependent preictal states.

The shifts in connectivity measured by BC or DC were also consistent at the individual seizure level. During the interictal period (both continuously modeled interictal data and the period from − 120 to − 38 s), there was no significant difference in DC between SOZ, PSZ, and NSZ contacts for any subject or seizure, nor any significant increase in SOZ, PSZ, or NSZ contacts during the entire interictal period associated with physiological events (e.g. arousals from sleep). During SOZ ∆ phase, DC rose significantly at SOZ channels in 138 of 143 (96.5%) seizure events (p < 0.05). In the PSZ/NSZ ∆ epoch, DC remained elevated or increased further in SOZ channels in 143 of 143 seizures (100%) and also increased significantly in both PSZ and NSZ channels in 136 of 143 seizures (95.1%, p < 0.05).

Similarly, BC at SOZ contacts in the SOZ ∆ epoch increased significantly (p < 0.05) in 141 of 143 seizures (98.6%). In the PSZ/NSZ ∆ phase, BC in PSZ nodes increased significantly in 132 of 143 seizures (92.3%), and BC in NSZ nodes showed an overall qualitative increase, which was significant in 120 out of 143 seizure events (83.9%). As with DC, there was no similar increase in BC in SOZ, PSZ, or NSZ contacts at any interictal time period.

We also assessed the effect of surgical outcome (ILAE Class I vs Class > I) on the centrality values of the generated networks by separating them into two groups and repeating the prior characterization. There was no significant difference in average degree or betweenness centrality between the two groups, although SOZ centrality values were qualitatively lower in Class > I cases in SOZ ∆ and PSZ/NSZ ∆ periods, resulting in changes in significance magnitude only.

### Localization and direction of preictal increased connectivity

**Figure 3:**
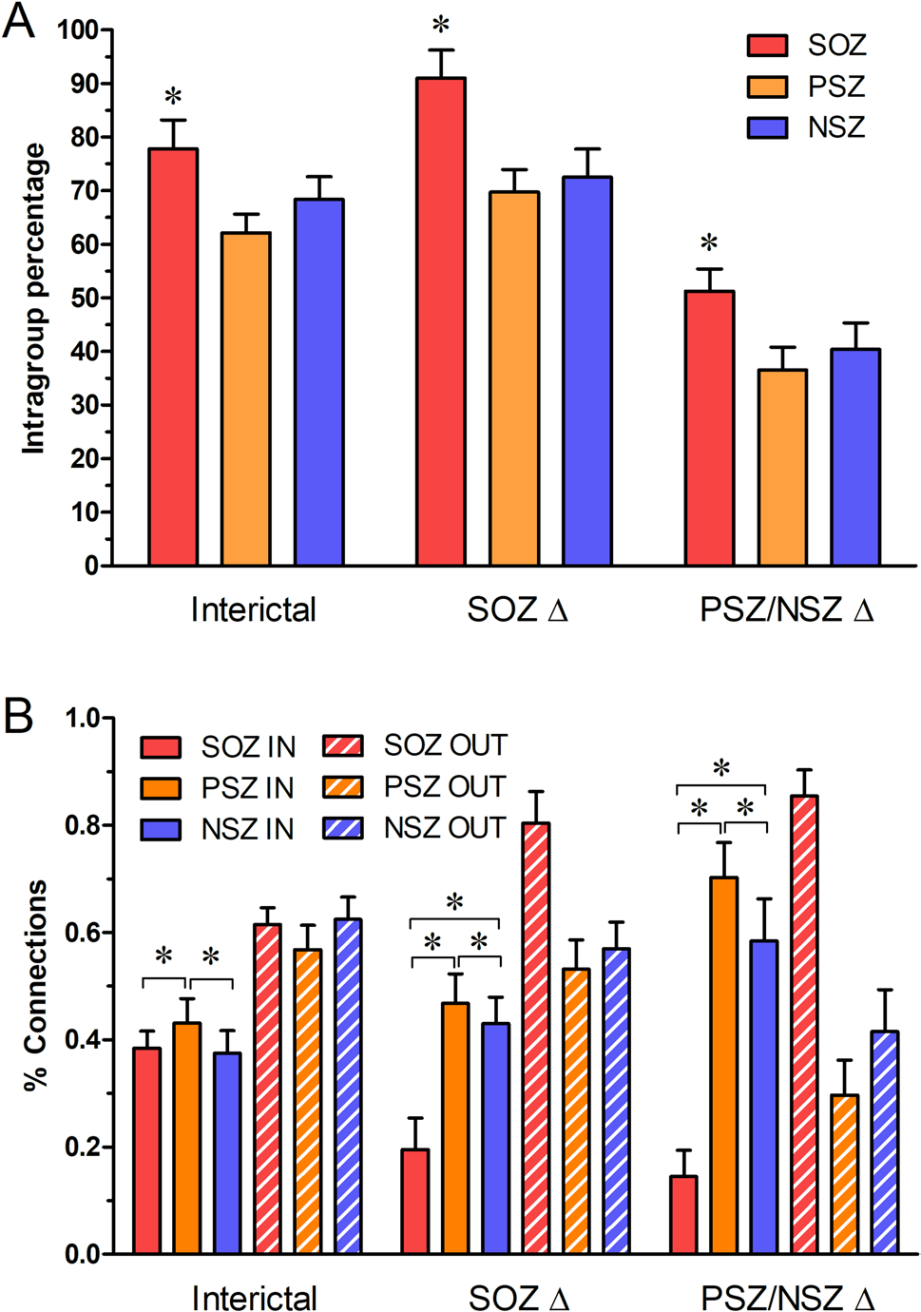
(**A**) Average percentage of intragroup network connections for each seizure over time in SOZ, PSZ and NSZ nodes during interictal, SOZ ∆, and PSZ/NSZ ∆ periods before seizure. Average percentage is reported as mean (solid bars) ± standard deviation (SD). Asterisks denote significant difference determined with Kruskal Wallis (p < 0.05) with post-hoc Dunn’s test. (**B**) Percentage of incoming vs outgoing directed network connections over time for SOZ, PSZ and NSZ channels during interictal, SOZ ∆, and PSZ/NSZ ∆ periods prior to seizure. Percent of incoming and outgoing connections is reported as mean, with hashed bars showing outgoing connections. Asterisks denote significant difference by Kruskal Wallis (p < 0.05) with post-hoc Dunn’s test.

To assess the possible functional role of these network changes in seizure onset, we asked whether connecting edges were located within the assigned node group (SOZ, PSZ or NSZ) or directed outward to nodes in one of the other groups. For each seizure, at every contact in the SOZ, PSZ, and NSZ groups, and at each of the 3 time periods, we labeled each connection as either intergroup or intragroup and determined the proportion of intergroup and intragroup connections for each node. During the interictal state, a significantly (p < 0.001) higher proportion of SOZ connections were within their group (77.8 ± 5.3% for SOZ compared to 62.2 ± 3.5% of PSZ and 68.4 ± 4.2% of NSZ contact connections), suggesting relative isolation of SOZ from PSZ and NSZ brain regions (Fig. 3A). During the SOZ ∆ period, the increase in connectivity among SOZ nodes is almost completely within SOZ, with intragroup connections significantly (p < 0.001) increased to 91.0 ± 5.2% of SOZ connections, while PSZ (69.7 ± 4.2%) and NSZ (72.6 ± 5.3%) remained relatively stable. This suggests increased isolation of SOZ from PSZ and NSZ nodes as connectivity in the SOZ increased. In the PSZ/NSZ ∆ period, the proportion of intragroup connections dropped dramatically, as SOZ and PSZ/NSZ electrodes became more broadly interconnected, with 51.3 ± 4.2% of SOZ, 36.6 ± 4.2% of PSZ, and 40.4 ± 5.0% of NSZ connections occurring within their group. Note that this increase in interconnection of SOZ and NSZ nodes occurred *before* the onset of electrographic seizure, not due to recruitment of PSZ/NSZ contacts during the seizure or resulting from electrographic seizure activity.

To identify the direction of influence or driving behavior within the network, we examined how the proportion of incoming vs outgoing directed connections at each node changed as a seizure approached. Incoming and outgoing connections are largely balanced in all three node location groups during the interictal state (Fig. 3B). During the SOZ ∆ phase, the proportion of outgoing connections in SOZ increases significantly (from 61.5 ± 3.2% to 80.5 ± 5.9%, p < 0.001), as a result of slightly decreased inputs from the surrounding PSZ and NSZ nodes as well as a decrease in their percent contribution relative to total SOZ connectivity, indicating increasing isolation of SOZ. Upon entering the PSZ/NSZ ∆ phase, there is a further significant increase in outgoing connections in SOZ nodes (to 85.5 ± 4.9%, p < 0.001), while significant increases in incoming connections occur in the PSZ (from 46.8 ± 5.5 to 70.3 ± 6.5%, p < 0.001) and NSZ (from 43.0 ± 5.0% to 58.5 ± 7.8%, p < 0.001) nodes. Together with the changes in intragroup-directed connectivity seen in Fig. 3A, this suggests that changes in incoming vs outgoing node connections during the PSZ/NSZ ∆ phase are driven by increased outgoing connectivity from or through hub nodes in SOZ.

**Figure 4:**
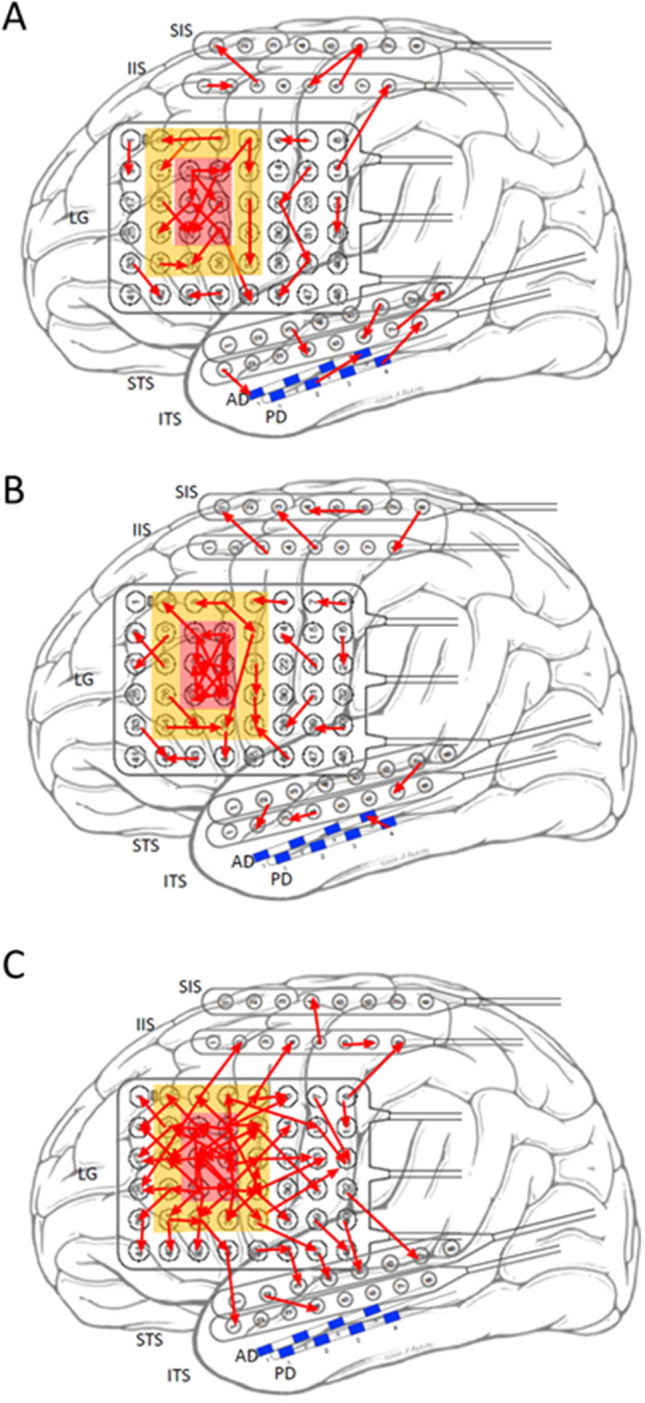
Directed graph visualization of network changes during the 3 preictal phases within 2 min of seizure onset for a single patient, mapped onto the patient’s grid placement diagram. Model shows only the top 1% strongest connections for clarity. SOZ electrodes are highlighted in red, PSZ in orange, and all others (NSZ) left uncolored. Red arrows represent directed connections. (**A**) Interictal connectivity graph shows low baseline connectivity and isolated connection clusters. (**B**) During the SOZ ∆ phase (− 37 to − 9 s) there is increased connectivity among SOZ contacts with sparse connections to outlying clusters. (**C**) During the PSZ/NSZ ∆ phase (− 8 s prior to seizure onset), greatly increased connectivity spreads to involve non-SOZ contacts.

A reduced connectivity model (using only the strongest 1% of connections rather than the 5% cut-off used in our analytical models) enables visualization of these network connections superimposed on the labeled grid for a single sample patient in each of the 3 putative network states (Fig. 4). During the interictal period (Fig. 4A) there is sparse overall connectivity with no particular relationship between SOZ, PSZ or NSZ. In the SOZ ∆ phase (Fig. 4B), interconnectivity within the SOZ intensifies, without affecting PSZ or NSZ regions. In the PSZ/NSZ ∆ phase (Fig. 4C), the intensified connectivity “breaks out” from the SOZ and spreads to PSZ and NSZ nodes, in the final 8 s prior to seizure onset.

### EEG frequency differences associated with preictal network state changes

**Figure 5:**
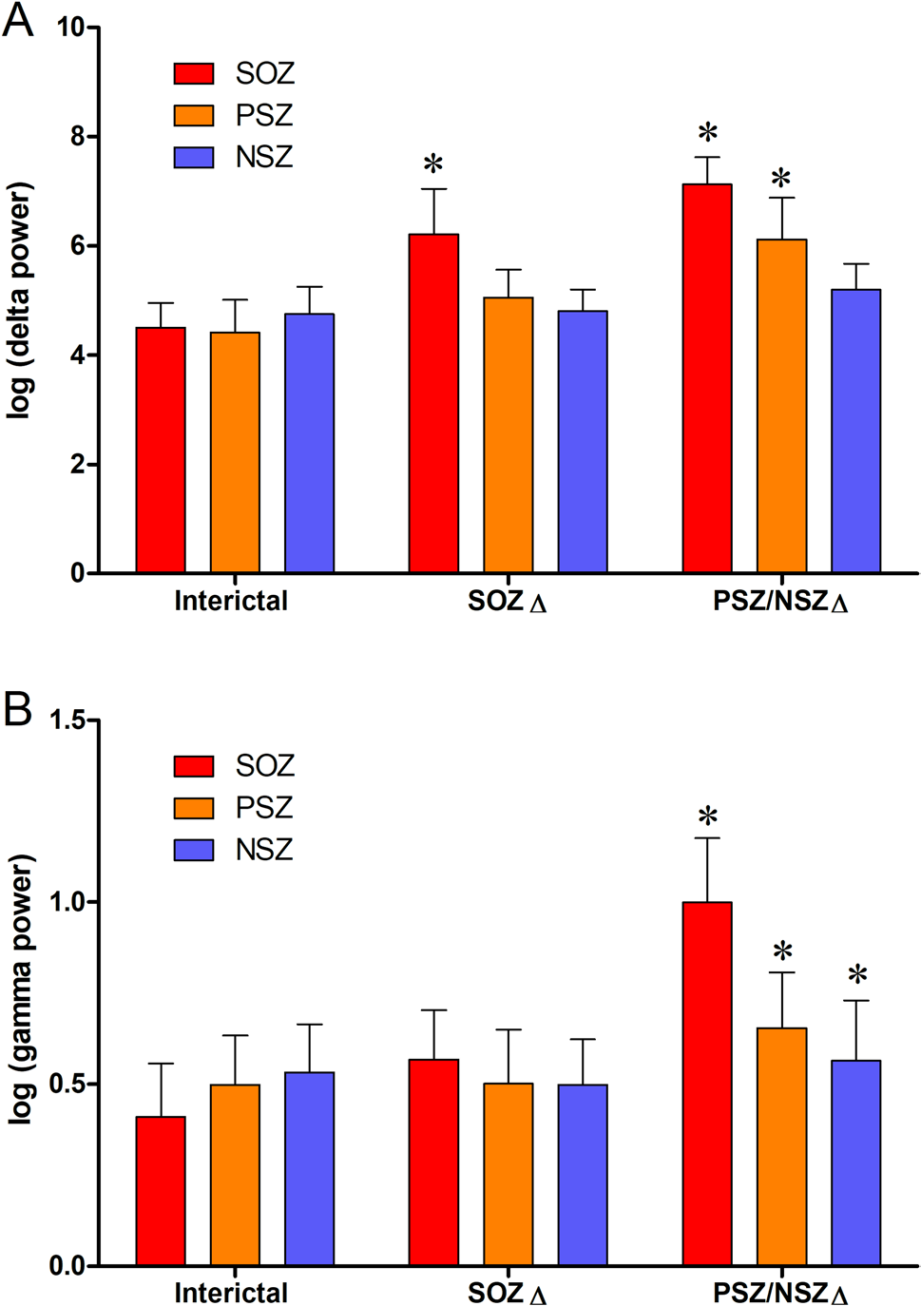
Regional canonical power band changes over time. (**A**) Average log (delta power) of SOZ, PSZ and NSZ channels during Interictal, SOZ ∆ and PSZ/NSZ ∆ periods before seizure. (**B**) Average log (gamma power) of SOZ, PSZ and NSZ channels during the same periods before seizure. Average is reported as mean (solid bars) ± SD (vertical bars). Asterisks denote significant difference relative to the interictal period for the same electrode region. Significant difference determined with ANOVA (p < 0.05).

To determine whether these network structural changes were associated with specific EEG frequency characteristics, we also examined the frequency components of SOZ, PSZ, and NSZ electrode activity, measuring power in the canonical (8–13 Hz alpha, 13–25 Hz beta, 4–7 Hz theta, < 4 Hz delta) frequency bands during each network state. Significant differences in power between node location groups or interictal and preictal periods were only seen in the delta and gamma frequency bands. As shown in Fig. 5A, SOZ contacts had significantly higher delta band power than both PSZ and NSZ contacts in the SOZ ∆ and PSZ/NSZ ∆ periods (p < 0.05), with PSZ delta power significantly increased over prior levels in the PSZ/NSZ ∆ period (p < 0.05, Fig. 5A). Gamma band power was significantly elevated at SOZ contacts in the PSZ/NSZ ∆ period (p < 0.05), with smaller but significant gamma power increases at PSZ and NSZ contacts (p < 0.05, Fig. 5B) during this period.

## Discussion

**Figure 6:**
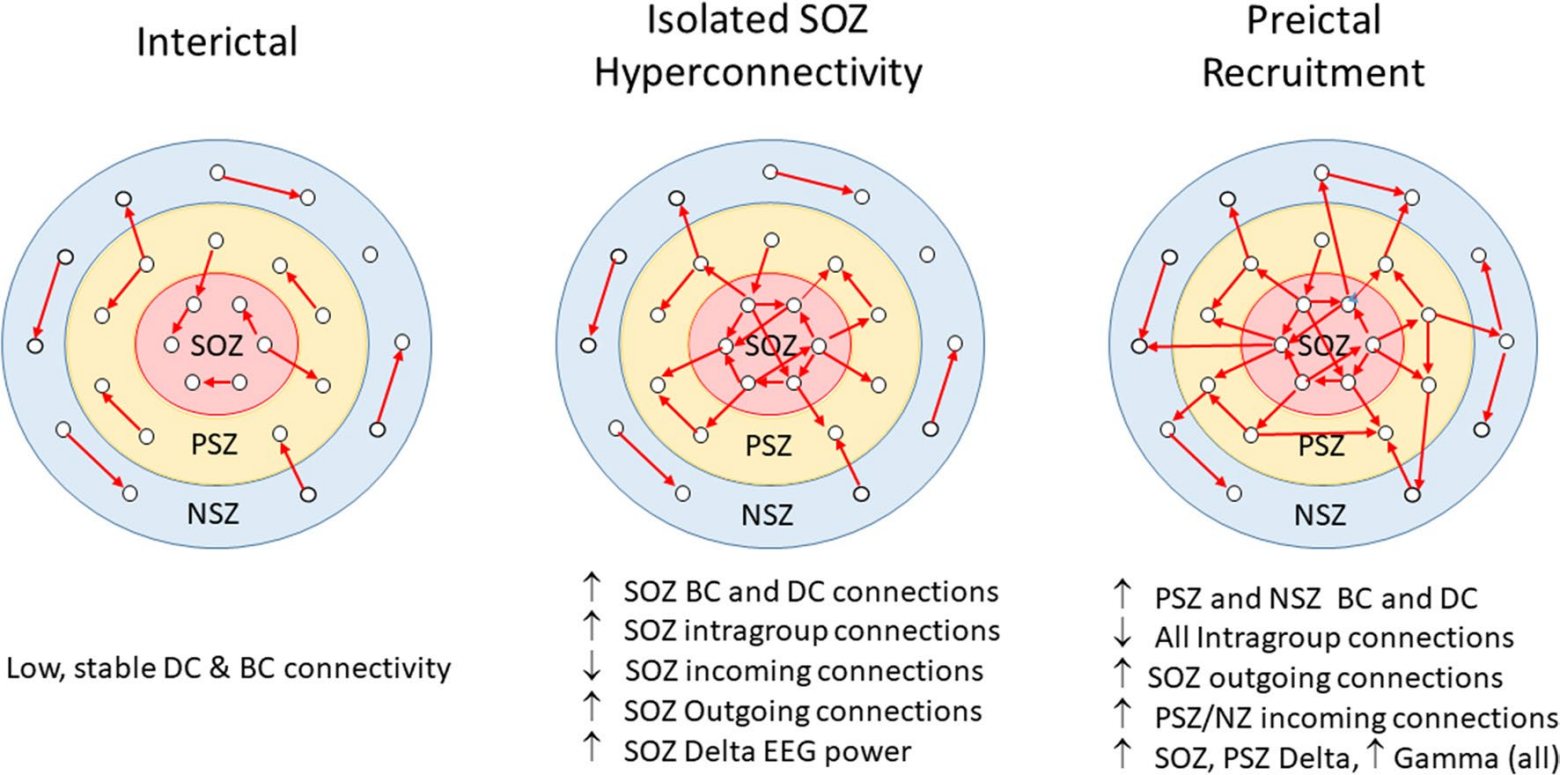
Diagram of preictal network state changes and the characteristics associated with each state. In this figure and the “Discussion”, we refer to the SOZ ∆ state as the “Isolated SOZ Hyperconnectivity” phase, and the PSZ/NSZ ∆ state as the “Preictal Recruitment” phase.

In this study, we analyzed dynamic network changes in the epileptic human brain over the 2 min period just prior to seizure onset in drug-resistant patients undergoing intracranial EEG monitoring. We hypothesized that the degree of network connectivity within the clinically defined SOZ and its connection to the surrounding tissue would vary significantly during the transition to a preictal state just prior to seizure onset. We implemented a novel application of multiple input, single output (MISO) state space modelling to identify meaningful predictive connections between brain regions with short duration epochs of iEEG recording, allowing a fine resolution estimation of network structure evolution over time. Network structure changes were assessed using graph theoretical tools measuring degree of interconnectedness and the extent to which information flow in the brain is dependent on specific nodes of the network. We found that the brain network transitions through 3 distinct states as a seizure approaches, and that the characteristics of those states are internally consistent until transition to the next state occurs (see diagram in Fig. 6). For interictal periods, both more than 2 h before and after a seizure and up to 1 min prior to seizure onset, gross network structure remained consistent with low and stable DC and BC connectivity, hence we classified both temporally removed epochs and the period up to about 40 s prior to seizure onset as the Interictal state. From 40 s prior to seizure onset until electrographic seizure detection, we observed two distinct network-state changes. First, a “transitional state” occurs with significantly increased degree and betweenness connectivity within SOZ, paired with a reduction in the proportion of connections from PSZ and NSZ to SOZ nodes and an increase in outgoing connections from SOZ. This suggests an internally generated increase in connectivity in the SOZ, relatively isolated from PSZ and NSZ influence, which begins to be directed outward but initially does not affect connectivity levels in these regions. At the same time, betweenness centrality in SOZ increased significantly and stably, suggesting that within SOZ itself certain “superhub” nodes are becoming more critical. Due to the increase in internal connectivity and relative isolation of SOZ, we now refer to this period as the “Isolated SOZ Hyperconnectivity” state (Fig. 6). Ictal activity was not detected at the macroscopic level at this time point, but asynchronous microseizures might explain the increased connectivity within smaller neuronal assemblies. The network changes within this state represent alterations in the effective network properties that are associated with, and hence may predispose toward, seizure onset, though the specific physiological events that cause them are uncertain. These changes persist until about 8 s before the clinically determined electrographic seizure onset, at which time there is a second state change. At this time, in all patients and most seizures, degree connectivity significantly increased for all recording nodes, and the proportion of connections between SOZ and surrounding regions increased significantly, with the majority of these new connections being directed connections from SOZ to PSZ and NSZ. We will now refer to this period as the “Pre-ictal Recruitment” state (Fig. 6). When the development of increased network connectivity projects out of SOZ and induces hyperconnectivity in PSZ and NSZ nodes, this appears to provide the necessary conditions for seizure onset and the subsequent appearance of electrographic seizure activity on clinical EEG.

Additionally, these changes in network structure are associated with changes in the power of specific EEG frequency bands, with SOZ delta power increasing in the Isolated SOZ Hyperconnectivity period and both delta and gamma power in the Preictal Recruitment period. Increased delta power in SOZ could indicate increasing synchrony of neuronal “up” and “down” states^21^ (as seen in slow wave sleep) and related to the synchronous GABAergic potentials that have been observed prior to seizure onset^22^. The transition from Isolated SOZ Hyperconnectivity to Preictal Recruitment might thus correlate with the failure of “inhibitory restraint” of epileptiform activity that occurs at seizure onset^23^. The increase in gamma band power in SOZ has previously been observed just prior to seizure onset^24–28^, and may have particular significance due to its potential to alter synaptic strength via short- or long-term potentiation^29–32^, which might explain the spread of connectivity from SOZ to PSZ and NSZ nodes.

Surprisingly, the changes in network structure that extend to PSZ and NSZ electrodes occur before the onset of the electrographic seizure, not during the seizure event as might be assumed based on the clinical EEG. This may seem contradictory to evidence at both macroscopic^33–35^ and microscopic^23,36^ levels as well as mathematical models^37^ demonstrating recruitment of epileptiform activity associated with seizure spread early in the course of a focal seizure. However, connectivity changes may reflect the creation of network connections sufficiently strong to allow the propagation of seizure activity, prior to the onset of actual epileptiform activity. This putative mechanism is remarkably consistent with the model proposed by Wenzel et al.^38^ in which seizures originate as hyperactive local neuronal ensembles within the initiation site (SOZ) which then engage larger areas in a saltatory fashion until the activity breaks into neighboring cortex, where it can be detected electrophysiologically as a local field potential. They proposed a two-step model for the progression of focal seizures in which neuronal ensembles first generate microseizures, followed by widespread neural activation in a traveling wave through neighboring cortex during “macro” (EEG electrographic) seizures. The “traveling wave” of seizure activity observed in both microscopic^36^ and macroscopic^23^ iEEG recordings may thus be propagated within the medium of increased connectivity established before the seizure begins. Since only macroscopic iEEG was available for the patients reported, we cannot address this hypothesis directly, but application of MISO models to microelectrode grid data might provide additional insight into seizure onset mechanisms.

An important contribution of this study is the network state transition explanation for seizure onset. Pathological activity begins in the SOZ, triggering a state change to a *transitional* state in which SOZ local activity becomes increasingly dependent on the activity of adjacent nodes and isolated from the rest of the brain. In this isolated state, local SOZ dependence intensifies, producing high interconnectivity and, ultimately, localized hyper-synchronization. This state builds in intensity, suggesting an underlying intrinsic driving process within SOZ. When this process reaches a critical point, a second state transition occurs, which we have termed the pre-ictal recruitment state. It then spreads outward, overwriting normal activity and manifesting as an electrographic seizure. Notably, this spread of dependence is detectable before clinically identifiable electrographic evidence, suggesting a network and pattern driven process, rather than a simple electrical “overwrite” or “recruitment” of independent rhythmic activity into the evolving rhythm of the seizure. This result contrasts with approaches that have focused on specific frequencies^39–43^, voltages^44–48^, or pathological^49–51^ causes, though the differences in delta and gamma power between SOZ and PSZ/NSZ contacts do suggest the possibility that certain frequency components may be involved. Because the patient pool for this study contains multiple different pathologies and etiologies, yet produces consistent results across all subjects, it is reasonable to suggest that while there may be a diverse array of dysfunctions that lead to the initial isolation of SOZ and generation of pathological activity, the process of focal seizure initiation and propagation may have consistent network-driven characteristics in many patients with focal epilepsy. In most cases, SOZ is also a region that generates interictal spike discharges^52–54^, associated with localized hyperexcitability due to recurrent excitation or loss of recurrent inhibition^55–58^, processes that could drive increased localized network connectivity^59^. However, the mechanisms underlying the observed network state transitions remain unclear.

From a clinical perspective, the high betweenness centrality (BC) of particular nodes within the SOZ indicates that a given node is critical to the information flow within the network. In the case of the isolated network identified in SOZ, our results suggest that BC within SOZ may be linked to hyperexcitable cortex necessary for seizure initiation and could potentially be used to target “seizure critical” or “superhub” nodes^60^ to maximize successful surgical outcomes with a smaller resected volume, or for stimulation using deep brain or responsive neurostimulation. Additionally, the dramatic changes in SOZ networks prior to seizure onset suggest an independent strategy for identification of SOZ nodes, which might be useful in surgical planning. This finding also implies that isolation of a local cortical network from surrounding networks may play a role in epileptogenesis. Further work is necessary to address these possibilities.

Several limitations of this study should be noted. The inclusion of patients who experienced both successful and unsuccessful surgeries as subjects raises potential issues regarding channel identification, since unsuccessful surgery suggests the possibility that channels may have been misidentified as non-SOZ resulting in lack of a surgical cure. Inclusion of “actual” SOZ channels in the PSZ or NSZ group could result in the “contamination” of the PSZ groups with channels better described as SOZ, or vice versa, despite the apparent lack of a significant overlap in results from SOZ and PSZ. However, given the robust findings of our analysis, the effect of such contamination appears to have been minimal. Moreover, a seizure free outcome does not guarantee that all of SOZ was resected; surgery may simply have eliminated critical nodes necessary for seizure initiation. Future investigations of pre-ictal network changes should evaluate a broader variety of patients and seizure localizations, including separate analysis of patients with unsuccessful surgeries. We did not stratify interictal EEG samples by patient state (wake vs. sleep) when the degree of network synchronization may differ, which might have altered network constructs, though our analysis of extended interictal EEG failed to identify any other physiologic events associated with similar increases in connectivity. Additionally, this analysis cannot be extended to generalized epilepsies, for which the underlying mechanisms may be quite different.

In summary, we find that two distinct network connectivity state changes occur in the final minute prior to the onset of focal seizures. This evidence suggests that seizure onset is associated not only with hyperexcitability and hypersynchrony, but also a third “H,” hyperconnectivity.

## Methods

### Dataset

All methods were carried out in accordance with relevant guidelines and regulations. The University of Connecticut Institutional Review Board considers retrospective research performed on data from fully de-identified patients as “Exempt Research,” which does not require IRB evaluation or approval. Twenty de-identified patients with drug-resistant epilepsy from the National Institutes of Neurological Disease and Stroke iEEG Portal (https://www.ieeg.org/)^61^ were included in this study. Information about the patients’ epilepsy etiology, iEEG recording setup, and surgical outcome are reported in Supplementary Table 1. Patients were selected based on three criteria, (i) a clinical report available on the iEEG Portal with information about the epilepsy etiology, type of seizure, and the clinically determined SOZ^62,63^; (ii) minimum duration of two continuous days of multichannel iEEG recording; and (iii) consistent sampling rate to enable homogenous analysis. All iEEG recordings were sampled at 512 Hz and patients were monitored for 2–7 consecutive days. The clinically determined SOZ channels were as marked by the original board-certified epileptologist who managed the patients, and were used as the standard for channel classification, irrespective of surgical outcome. Electrode contacts on the same grid, depth or strip electrode that were adjacent to SOZ electrodes were analyzed as peri-SOZ (PSZ), to assess changes in sampled brain regions neighboring the SOZ. All other electrode contacts were considered non-SOZ (NSZ). When more than one intracranial investigation was performed, only the final electrode placement and associated seizures were analyzed. A small number of seizures were excluded as too brief (< 15 s) for analysis.

Patient reports on the iEEG Portal describe a variety of seizure localizations and etiologies. Four patients had seizure onset within the temporal lobe, 5 in temporal lobe plus other neocortical regions, and 11 had extra-temporal epilepsy (10 frontal, 1 parietal). Eleven of 20 were left-hemisphere. Seven had a known etiology (3 meningitis, 3 traumatic brain injury, 1 dysplasia) and 13 had cryptogenic epilepsy. A total of 3382 h of continuous iEEG recordings were analyzed, an average of 169.1 ± 100.4 h (mean ± SD) per patient (min: 48 h; max: 314 h). Depth, strip and grid electrodes were placed according to the clinical hypothesis, with an average of 79.9 ± 17.2 electrode contacts per patient (range 52–104, see Supplementary Table S1 and Fig. S1). Assignments of electrodes to SOZ, PSZ and NSZ categories are listed in Supplementary Table S2. A total of 143 seizures were analyzed, an average of 7.2 ± 6.2 per patient (range 1–26 events). The clinically determined SOZ spanned an average of 8.15 ± 3.56 electrode contacts per patient (median = 7), which correspond to an average of 10.2 ± 3.9% of the number of electrode contacts per patient. An average of 12.75 ± 5.24 electrodes per patient were designated as Peri-SOZ (PSZ, 15.69 ± 5.78%). Twelve patients underwent epilepsy surgery, of whom 8 became seizure-free (Engel or ILAE Class 1 outcomes), 3 had significant improvement (ILAE Class 4) and one had no improvement (ILAE Class 5).

### EEG analysis

#### Preprocessing and network estimation

Individual iEEG time series were processed by 60 Hz notch filtering, band-pass filtering between 0.5 and 256 Hz with a 10th order Butterworth filter, and common average mode montage correction. For each seizure event, as identified from clinical notes, the two minutes prior to seizure was divided into a series of 120 ten-second epochs advanced with a sliding 1 s window for analysis. To ensure that other physiological events (e.g. arousals from sleep) did not also alter network structures, the entire interictal record for all patients (excluding the periods from 2 h before to 2 h after each seizure) was analyzed using the same method, a total of 163,082 distinct models covering 453 h or 18.9 days of recording. For each epoch, a directed network graph was estimated using multi-input, single-output state space models.

### Temporal evolution of EEG series

To estimate the epileptogenic effective network and quantify changes in the network connectivity over time, we envisioned the iEEG time series *y*_*k*_(*t*) at any contact *k*, with *k* = 1, 2, 3, …, *N*, as the output of a dynamical system driven by the activity at all iEEG contacts. We modeled each contact as a linear time-invariant multi-input, single-output system:

1 $$\begin{aligned} {{\dot{\mathbf{x}}}_{k}} &= {\mathbf{A}}_{k} {\mathbf{x}}_{k} (t) + {\mathbf{B}}_{k} {\mathbf{Y}}(t) \\ {y_{k}} (t) &= {{\mathbf{C}}_{k} {\mathbf{x}}_{k} (t) + e_{k} (t)} \\ \end{aligned}$$

where **x**_*k*_(*t*) is a *m*-dimensional column that captures the internal state of the brain region sampled by the iEEG contact *k* at time *t*, **Y**(*t*) is the 1 × *N* row vector of iEEG samples at time *t* (one sample per contact), *e*_*k*_ is measurement noise (assumed to be a Gaussian process with zero-mean), and matrices **A**_*k*_ (size: *m* × *m*), **B**_*k*_ (size: *m* × *N*), and **C**_*k*_ (size: 1 × *m*) are parameters to be estimated.

The estimation of parameters (**A**_*k*_, **B**_*k*_,** C**_*k*_,) was conducted separately for every iEEG contact *k* during each epoch using a least-squares method with QR factorization for multiple values of *m* using a cross-validation method, i.e., for each epoch and iEEG contact, parameters were estimated on the first 80% (8 s) of the iEEG time series of interest and the goodness-of-fit of the resultant model was measured on the remaining 20% (last 2 s of the 10 s window). A model was accepted and estimated parameters were used if the residuals *y*_*k*_(*t*) – **C**_*k*_**x**_*k*_(*t*) estimated for the test data passed the test for whiteness and independence at 95% confidence (*p* < 0.05). Since the size *m* of the internal state **x**_*k*_(*t*) determines the complexity of model (1), and high values of *m* may result in data overfitting, we finally chose *m* by minimizing the average Akaike Information Criterion (AIC)^64^ index estimated across all iEEG contacts, epochs, and conditions. Models of identical size were used for every contact, epoch, or condition, facilitating comparison while preventing overfitting.

### Evaluation of B matrices to determine network connectivity

Parameters for the MISO SS model are found by iterative minimization of next-step prediction error, resulting in a model that accurately represents the electrographic behavior of the system based on its internal state matrix **A**_*k*_, previous timepoint activity **x**_*k*_(*t*), and the weighted **B**_*k*_ influence of the activity at all other electrodes **Y**(*t*). We then can interrogate relationships in the system by examining the parameter matrices. In particular, the input matrix **B**_*k*_ represents a set of weighting factors that describes the extent to which next-step *k* + *1* activity at the given electrode is dependent on the activity of corresponding entries in the input vector **Y**(*t*). It follows that high magnitude values in the input matrix **B**_*k*_ are associated with specific input vectors (activity at other electrodes) that have a high degree of influence on the electrographic output at the given channel. From this, we can infer that these channels have a significant causal/driving connection to the given output channel. This relationship can be expressed as a simple network of directed connections to that channel. We then repeat the MISO SS model estimation for each iEEG channel independently, resulting in a set of simple, single sink networks capturing the causal connections to that sink, the output channel of interest. By combining each output channel network into a unified network that includes all of the sampled channels, we can fully capture the directed causal connections in the recorded area, producing an effective connectivity network for a given time point. By iterating this process over time, we generate a time-varying effective network, which can be quantified at each time point by graph theoretical analysis.

For every condition, matrices **B**_*k*_, *k* = 1, 2, 3,…, *N* in (1) were used to define the connectivity between contacts and retrieve the current effective network. Specifically, the Euclidean norm $b_{kj} \triangleq\| {{\mathbf{b}}_{k,j} } \|_{2}$ of column vector **b**_*k,j*_ measured the magnitude of the influence of contact *j* on the activity (or the internal state driving the activity) at contact *k* and was used as a measure of the effective connectivity from *j* to *k* ($j \to k$). Similarly, *b*_*jk*_ was used as a measure of the effective connectivity from contact *k* to contact *j* ($k \to j$) and resulted in $b_{jk} \ne b_{kj}$. The *N* × *N* matrix ${\hat{\mathbf{B}}} =\{ {b_{ij} } \}_{1 \le i,j \le N}$ was non-symmetric and defined the oriented graph that characterizes the brain network captured by the magnitude of directed influence *b*_*kj*_. Sequential 10-s windows sliding at 1 s intervals were used to estimate ${\hat{\mathbf{B}}}$ matrices for the two minute periods prior to each seizure event (120 separate models per seizure) and additional models were estimated at 1 s intervals for the entire interictal period excluding the two hours before and after each seizure. Matrices ${\hat{\mathbf{B}}}$ were pruned of nonsignificant elements by computing the sample probability distribution function of values *b*_*kj*_ across all matrices ${\hat{\mathbf{B}}}$ for all patients and retaining those values above the 95th percentile, while the remaining values were set to zero. After pruning, matrices ${\hat{\mathbf{B}}}$ were generally sparse and retained strong links between nodes, with a clear indication of the nodes that drove the network evolution in each condition. Larger values of *b*_*kj*_ indicate a larger influence of the corresponding node. Relative changes in connection strength between nodes were not assessed, since only strong connections were included in final connectivity graphs.

### Network analysis

The topology of the oriented networks was used to assess the effects of the approaching seizure on network structure. The topology was quantified using two metrics: degree centrality (DC) and betweenness centrality (BC). DC and BC measure the average density of connections to a node (DC) and the number of hubs in the network (BC), respectively. Accordingly, higher values of DC or BC indicate networks whose nodes are more densely connected (DC) or networks whose nodes are largely connected through a handful of nodes (i.e., hubs) that receive many connections and project onto many nodes (BC), respectively. Using the directed nature of the connections estimated with our method, we calculated the proportion of connections that were incoming vs outgoing from each node, separated by channel group, to identify changes in whether a group was driving or being driven by the activity of other nodes. Additionally, to determine whether observed state changes were associated with overt changes in the EEG frequencies recorded at SOZ or other electrodes, we used Fast Fourier Transform (FFT) to analyze power of canonical frequency bands at SOZ nodes vs PSZ and NSZ nodes during three preictal time periods found to have distinct network characteristics suggesting differing network states, as reported.

### Statistical analysis

Statistical differences for DC and BC were determined using the nonparametric Kruskal–Wallis test with Dunn’s multiple comparisons post-hoc testing and rejected at the 95% confidence level (*p* < 0.05), where the preceding epoch/control epoch and contact type (SOZ or NSZ) were the variables. Preprocessing routines, model estimation, network, and classification analysis were computed in MATLAB, ver. 2021b (Mathworks, Natick, MA), implemented using locally developed programming, with additional statistical analysis and figures produced using Prizm 5.0 (Graphpad Software, San Diego, CA).

## Supplementary Information

Supplementary Information.

Supplementary Figure S2.

## Data Availability

All data analyzed for this paper is publicly available on the iEEG Portal (https://www.ieeg.org/). This site requires the user to create an account and state the purpose of their interest in the data. There are no restrictions to access after a login account has been created. The patient identification numbers are unchanged from those used on the iEEG portal website.
