# Supplementary material for: Network analysis of preictal iEEG reveals changes in network structure preceding seizure onset
Source: Sci Rep. 2022 Jul 22;12:12526. doi: 10.1038/s41598-022-16877-x (PMC9307526; doi:10.1038/s41598-022-16877-x)
Supplement: Supplementary file 1 — Supplementary Information. [file 41598_2022_16877_MOESM1_ESM.docx]

**Supplementary Materials:**

| **SUPPLEMENTARY TABLE I** | | | | | | | | | | | | | | | |  |
| --- | --- | --- | --- | --- | --- | --- | --- | --- | --- | --- | --- | --- | --- | --- | --- | --- |
| **Patient ID** | **Sex/**  **Age (y)** | | **Seizure Onset Zone** | | **Etiology** | | **Seizure Type** | | **No. of Seizures** | | **iEEG electrodes (depth/strip/grid)** | | **iEEG recording time (h)** | | **Post-surgery Outcome** | |
| Study_006 | M / 25 | Left Frontal | | Cryptogenic | | CP | | 5 | | 0 / 8 / 48 | | 27 | | *n/a* | |  |
| Study_010 | F / 13 | Left Frontal | | Cryptogenic | | CP / GTC | | 2 | | 0 / 8 / 48 | | 304 | | *n/a* | |  |
| Study_011 | F / 34 | Right Temporal | | Cryptogenic | | CP | | 3 | | 0 / 36 / 48 | | 84 | | *n/a* | |  |
| Study_012-2 | M /33 | Right Temporal Orbitofrontal | | Cryptogenic | | CP / GTC | | 26 | | 0 / 24 / 60 | | 277 | | ILAE-I | |  |
| Study_014 | F / 33 | Left Frontal | | Cryptogenic | | CP | | 8 | | 0 / 16 / 88 | | 136 | | *n/a* | |  |
| Study_016 | F / 36 | Right Temporal Orbitofrontal | | Meningitis | | CP / GTC | | 3 | | 0 / 16 / 48 | | 141 | | ILAE-IV | |  |
| Study_019 | F / 33 | Left Temporal | | Cryptogenic | | CP / GTC | | 14 | | 8 / 28 / 60 | | 136 | | ILAE-V | |  |
| Study_020 | M / 10 | Right Frontal | | Cryptogenic | | CP / GTC | | 8 | | 0 / 16 / 40 | | 120 | | ILAE-IV | |  |
| Study_021 | M / 16 | Right Temporal Orbitofrontal | | Cryptogenic | | CP / GTC | | 11 | | 0 / 8 / 96 | | 155 | | ILAE-I | |  |
| Study_023 | M / 16 | Left Temporal-Occipital | | TBI | | CP | | 3 | | 8 / 16 / 64 | | 48 | | ILAE-I | |  |
| Study_026 | M / 9 | Left Frontal | | Cryptogenic | | CP | | 17 | | 0 / 32 / 64 | | 70 | | ILAE-I | |  |
| Study_028 | M / 5 | Left Parietal | | Cryptogenic | | GTC | | 3 | | 0 / 16 / 80 | | 40 | | ILAE-IV | |  |
| Study_037 | F / 62 | Right Frontal | | TBI | | CP | | 8 | | 0 / 16 / 64 | | 215 | | *n/a* | |  |
| Study_038 | M / 58 | Left hippocampus, L mediofrontal | | TBI | | CP /  GTC | | 9 | | 8 / 32 / 48 | | 88 | | ILAE-I | |  |
| HUP_64 | M / 20 | Left Frontal | | Dysplasia | | SP / GTC | | 1 | | 0 / 24 / 68 | | 307 | | ENGEL-I | |  |
| HUP_65 | M / 36 | Right Temporal | | Cryptogenic | | CP | | 3 | | 0 / 16 / 64 | | 304 | | *n/a* | |  |
| HUP_68 | F / 26 | Right Temporal | | Meningitis | | CP / GTC | | 5 | | 0 / 24 / 64 | | 310 | | ENGEL-I | |  |
| HUP_70 | M / 32 | Left Perirolandic | | Cryptogenic | | SP | | 8 | | 0 / 14 / 64 | | 160 | | *n/a* | |  |
| HUP_72 | F / 27 | Left Frontal | | Cryptogenic | | GTC | | 1 | | 0 / 52 / 0 | | 314 | | *n/a* | |  |
| HUP_73 | M / 39 | Anterior Right Frontal | | Meningitis | | CP / GTC | | 5 | | 0 / 56 / 0 | | 146 | | ENGEL-I | |  |

| Patient ID= unique identifier in the iEEG Portal database (Azarion *et al*., *Epilepsia*, 55:2028-37, 2014). TBI= traumatic brain injury. CP= complex partial seizure. GTC= generalized tonic-clonic seizure. SP= simple partial seizure. *n/a*= Patient did not undergo surgery.  ------------------------------------------------------------------------------------------------------------------------------------------------- |
| --- |
|  |
|  |

**Supplementary Table 2: Electrodes Designations as SOZ and PSZ**

| **Map*** | **Patient ID** | **SOZ electrodes** | **PSZ electrodes** |
| --- | --- | --- | --- |
| A | Study_006 | LG: 7, 15, 16, 23, 24 | LG: 6, 8, 14, 22, 30-32 |
| B | Study_010 | LG: 6, 7, 13, 14 | LG: 4, 5, 8, 12, 15, 16, 20-24 |
| C | Study_011 | IA: 2, 3; IP: 3, 4; RG: 44, 45 | IA: 1, 4; IP: 2; RG: 35-38, 43, 46 |
| D | Study_012-2 | LG: 8, 9, 14, 15; RMT:2-7; RIT: 2-7 | LG: 1-4 7, 10, 13, 16, 19-22; RMT: 1, 8; RIT: 1,8 |
| E | Study_014 | LFG: 18-20, 26-28, 35, 36 | LFG 9-13, 17, 21, 25, 29, 33, 34, 37, 42-45 |
| F | Study_016 | RTG: 1, 5, 9, 22; ROF: 2-4 | RTG: 2, 6, 10, 13, 14, 17-19, 21, 23; ROF: 1 |
| G | Study_019 | LT: 1-3, 7, 15, 16, 21, 22 | LT: 4, 8-10, 13, 14, 17, 19, 20, 23, 26-29 |
| H | Study_020 | RAG: 7-10, 14-16 | RAG: 1-5, 11, 13, 17, 19-23 |
| I | Study_021 | RFG: 41-44, 46-48; RAT: 2-3 | RFG: 33-40, 45; RAT: 1, 4 |
| J | Study_023 | LTG: 10, 11, 18 , 19, 58, 59 | LTG: 1-4, 9, 12, 17, 20, 25-28, 49-52, 57, 60 |
| K | Study_026 | LFG 15, 24, 29, 30, 33, 34, 41, 46, 51-54, 58-61 | LFG 6-8, 14, 16, 21- 23, 25-27, 29, 32, 35, 42-47, 49, 50, 55, 57, 62 |
| L | Study_028 | LPG: 44, 51-53, 61 | LPG: 42, 43, 45, 46, 50, 54, 58-62 |
| M | Study_037 | RFS: 2; RPG: 35-37, 43-45 ,62-64 | RFS: 1, 3; RPG: 26-30, 34, 38, 42, 46, 50-56, 61 |
| N | Study_038 | LG: 11, 12, 19, 20, 27, 28 | LG: 2-5, 10, 13, 18, 21, 26, 29, 34-37 |
| O | HUP_64 | LG: 15, 22, 23, 30, 31, 38, 39 | LG: 6-8, 13, 14, 16, 21, 24, 29, 30, 32,37, 45-48 |
| P | HUP_65 | RG: 1-7, 9, 10, 17, 18; RAT: 2-4 | RG: 11-16, 19, 25-27; RAT: 1 |
| Q | HUP_68 | RG: 2-4, 10-14; RST: 3-4 | RG: 1, 5-7, 9, 15, 17-23; RST: 2 |
| R | HUP_70 | LG: 12-15, 20-23, 28, 29 | LG: 3-8, 11, 16, 19, 24, 27, 40, 35-38 |
| S | HUP_72 | LPP: 3-6 | LPP: 1-2 |
| T | HUP_73 | ROF: 1-5 | ROF: 6-8 |

* See Figure S1 with maps of electrode placements.

Supplementary Figure S1.


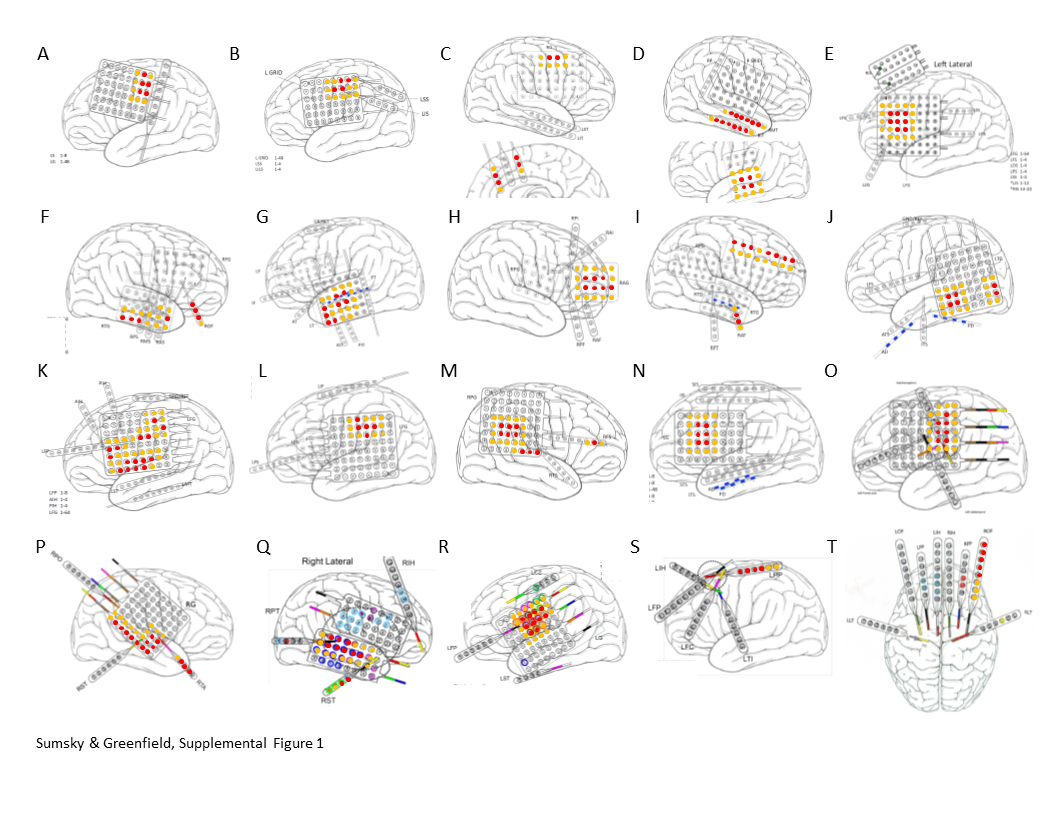


**Supplemental Figure S1**: Electrode placement diagrams from the iEEG Portal. Electrodes marked in red are designated as SOZ, and electrodes in orange are PSZ. A. Study006. B. Study010. C. Study011 (uninvolved mesial strips not shown) D. Study012. E. Study014. F. Study_016. G. Study019 H. Study020. I. Study021. J. Study023. K. Study026. L. Study028. M. Study037. N. Study038. O. HUP064 (uninvolved LH strip not shown). P. HUP065 Q. HUP 68 (uninvolved LH strip not shown). R. HUP070. S. HUP072 (mirror image strip placement over RH not shown). T. HUP073.
