# Supplementary material for: Network analysis of preictal iEEG reveals changes in network structure preceding seizure onset
Source: Sci Rep. 2022 Jul 22;12:12526. doi: 10.1038/s41598-022-16877-x (PMC9307526; doi:10.1038/s41598-022-16877-x)

Supplemental Figure S2: Generation of Effective Networks using MISO State Space Models.

A. Intracranial electrode recordings generate time-series data (*y*(t)) for each electrode channel. Five channels are shown for illustration. B. For each output contact (here contact 1 is selected) the State Space equation is applied to generate models of the activity at the target electrode by minimizing the prediction error of the state space matrix parameters (A) and the input matrix parameters (B) applied to all other electrodes. C. After iterative modeling of the first 8 seconds of each 10 second window, the parameters are tested for predictive value of the remaining 2 seconds. When an accurate fit is achieved, the B matrix parameters are used to determine which input electrodes have the highest influence on the output channel. Sequential application to each electrode as the output channel allows construction of a complete network graph at that time point.


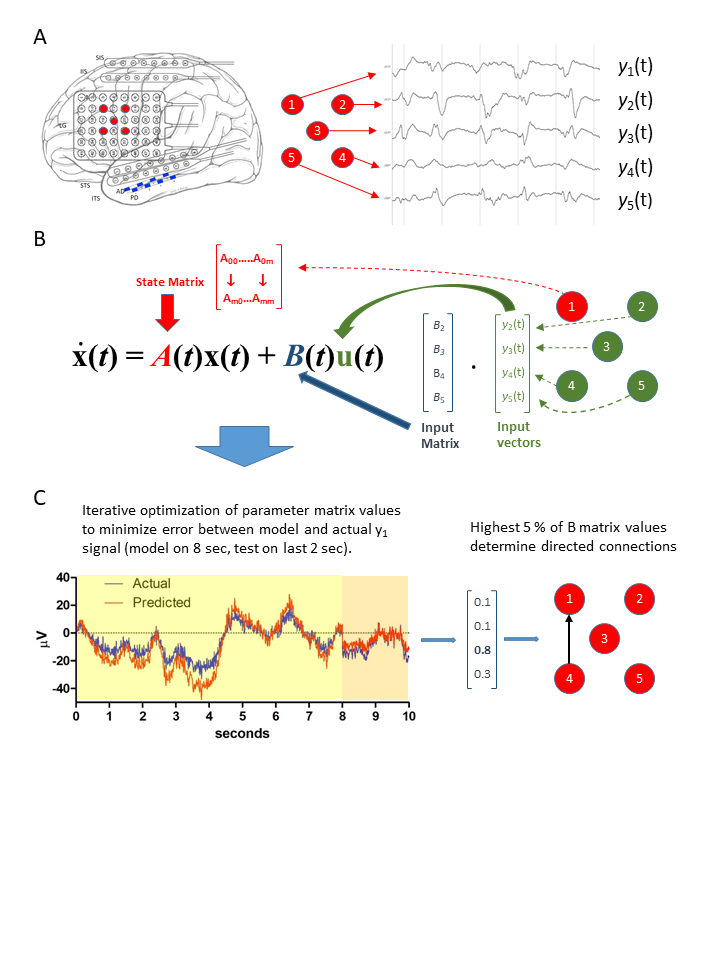

Supplement: Supplementary file 2 — Supplementary Figure S2. [file 41598_2022_16877_MOESM2_ESM.docx]
